# Arcuate fasciculus cortico-cortical and axono-cortical evoked potentials with post-resection excitability changes in perisylvian glioma

**DOI:** 10.1016/j.cnp.2026.07.013

**Published:** 2026-07-21

**Authors:** Ryan P. Hamer, Andrew Morokoff, Chris Donaldson, Kristian Bulluss, Andrew Gogos, Mohammed Awad, Santosh Poonnoose, Ahmad Badran, Adrian Praeger

**Affiliations:** a Department of Neurosurgery, St Vincent's Hospital Melbourne, Melbourne, Victoria, Australia; b Melbourne Medical School, University of Melbourne, Melbourne, Victoria, Australia; c Faculty of Medicine & Health, University of Sydney, Sydney, New South Wales, Australia; d Department of Neurosurgery, Royal Melbourne Hospital, Melbourne, Victoria, Australia; e Department of Neurosurgery, Austin Hospital, Melbourne, Victoria, Australia; f Flinders Health and Medical, Research Institute, College of Medicine and Public Health, Flinders University, Adelaide, Australia; g Department of Neurosurgery, Flinders Medical Centre, Bedford Park, South Australia, Australia; h Department of Neurosurgery, Monash Health, Melbourne, Victoria, Australia; i Department of Surgery, School of Clinical Sciences at Monash Health, Monash University, Melbourne, Victoria, Australia

**Keywords:** Arcuate fasciculus, Cortico-cortical evoked potentials, Axono-cortical evoked potentials, Language mapping, Glioma surgery, Intraoperative neurophysiological monitoring

## Abstract

**Objective:**

To evaluate the feasibility and clinical utility of arcuate fasciculus cortico-cortical evoked potentials (AF-CCEP) and axono-cortical evoked potentials (AF-ACEP) during perisylvian supratentorial tumor surgery, and examine whether morphology changes serve as electrophysiological biomarkers of postoperative language function.

**Methods:**

We analysed 19 patients with tumors involving or adjacent to the arcuate fasciculus across multiple tertiary centers. AF-CCEPs were elicited by monophasic stimulation toward the inferior frontal gyrus and recorded over the middle or posterior superior temporal gyrus; AF-ACEPs were obtained via subcortical bipolar stimulation. Primary outcomes explored N1 morphology pre- and post-resection, with subgroup analyses by tumor grade, location, montage, and anesthesia.

**Results:**

822 AF-CCEPs and 134 AF-ACEPs were acquired intraoperatively; 133 and 37 matched recordings were included for analysis, respectively. Post-resection N1 peak amplitude increased cohort-wide, while onset latency remained stable. One patient demonstrated signal loss associated with postoperative aphasia. AF-ACEP amplitudes were higher under general anesthesia, in higher-grade tumors, and with proximity to the AF on tractography.

**Conclusions:**

AF-CCEP and AF-ACEP are feasible intraoperative adjuncts, with signal degradation associated with language morbidity. Post-resection amplitude increases may reflect intact AF conduction with increased cortical excitability following tumor removal.

**Significance:**

When awake craniotomy is contraindicated, AF-CCEP and AF-ACEP may represent a viable alternative for monitoring AF integrity and reducing postoperative language morbidity.

## Highlights

• Cortico-cortical N1 peak-amplitude signal loss associated with post-operative aphasia.
• Post-resection N1 peak-amplitude changes observed in frontal and temporal tumors.
• Axono-cortical amplitudes higher under general anesthesia and higher-grade gliomas.

## 1 Introduction

The classical Broca-Wernicke-Geschwind model has long served as a foundational framework for understanding the cortical organization of language, which posits that Broca's area involving the pars opercularis of the dominant inferior frontal gyrus mediates language production, and Wernicke's area involving the posterior superior temporal gyrus and adjacent angular gyrus subserves language comprehension. The arcuate fasciculus (AF) is a frontotemporal association fiber tract that integrates expressive language function with receptive language, acting as a critical conduit linking these regions and forming a critical component of the dorsal language pathway (Geschwind, 1970; Catani et al., 2005; Dick and Tremblay, 2012).

The importance of Broca's and Wernicke's areas as central language hubs is frequently assumed a priori in the context of surgical planning, despite increasing evidence of inter-individual variability suggestive of a greater distribution of cortical-subcortical language networks. Although considered reductionist homologues, they are still often referenced within neurosurgical nomenclature and remain central to operative planning. Indeed, advances in neuroimaging and intraoperative electrophysiological mapping have since demonstrated that this serial, localist view is overly simplistic, giving rise to more nuanced models that account for the distributed and parallel nature of language processing comprising dorsal and ventral pathways (Hickok and Poeppel, 2007; Saur et al., 2008). This evolving paradigm emphasizes the integrated role of cortical and subcortical pathways in language, informing neurosurgical planning for tumors involving or suspected to involve eloquent cortex.

In patients harboring supratentorial perisylvian glioma, disruption of the dominant AF during resection can contribute to profound postoperative language deficits, most notably conduction aphasia and impaired verbal repetition (Duffau et al., 2008). However, language related deficits following surgery are often reported to be transient, provided that underlying white matter tracts are preserved (Duffau et al., 2008). Given its deep subcortical course and proximity to eloquent cortical regions, preoperative delineation and preservation of the AF are paramount to reducing the risk of iatrogenic injury contributing to devastating language deficits (Sarubbo et al., 2016).

Functional neuroimaging modalities such as functional Magnetic Resonance Imaging (fMRI), and Navigated Transcranial Magnetic Stimulation (nTMS) are considered contemporary risk stratification tools intended to promote opportunities of safe and maximal resection with preservation of language function. Each modality is limited in contrast to the ‘gold standard’ low-frequency bipolar Direct Cortico-Subcortical Stimulation (DCS), which is capable of functionally confirming proximity to vital language pathways following systematic stimulation mapping and subsequent transient disruption of language tasks during awake craniotomy. Comparative studies further indicate that nTMS may outperform fMRI in sensitivity and negative predictive value for language localization, particularly when awake mapping is contraindicated (Muir et al., 2022; Tarapore et al., 2013; Ille et al., 2024). These are vital pre-operative investigations that attempt to individually-tailor operative strategies to the patient, reflecting a growing understanding of hodotopic language networks in mono- and multilingual patients (Vilasboas et al., 2017) and functional reorganization as a consequence of glioma-induced neuroplastic potential (Ng et al., 2023).

Similarly, anatomic connectivity can be demonstrated via diffusion tensor imaging (DTI) in vivo. While DTI identifies similarly oriented myelinated axonal pathways, it does not necessarily indicate functional or effective communication between brain regions (Catani et al., 2002), though it has become an essential adjunct in neurosurgical planning. Commercially available DTI platforms permit the visualization of major subcortical white matter language pathways, including the AF, inferior fronto-occipital fasciculus (IFOF), superior longitudinal fasciculus (SLF-III), frontal aslant tract (FAT) and others. However, these methods often rely on anatomical landmarks or atlas-based regions of interest, which can be displaced or distorted by tumor-related mass effect or oedema, limiting functional precision and relevance. In contrast, nTMS-seeded DTI integrates individualized functional data derived from preoperative nTMS, which can enhance anatomical fidelity and improve the specificity of tract reconstruction (Ille et al., 2018). This approach has demonstrated superior correlation with intraoperative DCS, improved risk stratification, and increased predictive value of post-operative functional outcomes in both motor and language domains (Ille et al., 2018; Sollmann et al., 2020).

Early surgical intervention and safe maximal resection with DCS is associated with reduced incidence of iatrogenic and neurologic morbidity (Rossi et al., 2021; De Witt Hamer et al., 2012), improved quality of life, and opportunities for progression-free survival and long-term functional independence (Hervey-Jumper and Berger, 2015). Intraoperative DCS mapping techniques partnered with neuropsychological assessment are considered a standard of care to confirm cortico-subcortical language eloquence (Duffau et al., 2005; Sanai et al., 2008), and also serves to promote opportunities of maximal safe resection, prognostically quantify neurologic function, and inform subsequent post-operative management. Multimodal electrophysiological mapping and monitoring workflows, including monopolar high-frequency and bipolar low-frequency DCS, are commonly utilized in-tandem in neuro-oncology surgery (Hamer et al., 2020).

Cortico-Cortical Evoked Potentials (CCEPs) are electrophysiological responses elicited by direct electrical stimulation of one cortical region and recorded at another functionally connected cortical site. This technique enables the in vivo assessment of effective connectivity between distinct cortical areas by analyzing the time-locked propagation of electrical activity through white matter pathways. Regarding the arcuate fasciculus, AF-CCEP permits continuous intraoperative monitoring for the detection of iatrogenic injury associated with postoperative language deficits. For the measurement of proximity to white matter fibers of the AF within the resection cavity, Axono-Cortical Evoked Potentials (AF-ACEP) can be conducted to reduce the likelihood of iatrogenic dissection. Originally reported by Matsumoto et al. (2004), AF-CCEPs have since emerged as a viable but investigational adjunct to contemporary brain mapping techniques in neuro-oncology surgery (Yamao et al., 2017; Yamao et al., 2014; Saito et al., 2014; Yamao et al., 2021; Nakae et al., 2020; Giampiccolo et al., 2021; Saito et al., 2022; Vega-Zelaya et al., 2023; Seidel et al., 2024; Kamada et al., 2025; Kim et al., 2022), though formal clinical validation and consensus guidelines have yet to be established.

AF-CCEPs can be implemented in two directions subject to the site of stimulation. The first involves stimulating anteriorly involving the pars opercularis of the inferior frontal gyrus and recording posteriorly at the middle or posterior superior temporal gyrus, while the alternative entails the inverse. Due to the widespread distribution of AF projection fibers throughout the temporal lobe, the anterior-to-posterior method appears to offer greater anatomical specificity and is generally more clearly defined than the posterior-to-anterior approach (Seidel et al., 2024; Kamada et al., 2025).

Whilst data supporting clinical utility of AF-CCEP is highly encouraging, awake craniotomy with DCS remains the standard of care for language-eloquent mapping in glioma surgery due to its capacity for real-time functional assessment through direct patient interaction (Duffau, 2005; Sanai and Berger, 2010). However, several clinical factors can limit its feasibility. These include extensive tumor burden, advanced age, psychological intolerance, and increased risk of intraoperative seizures (Nossek et al., 2013; Hervey-Jumper et al., 2015). Preoperative language impairment may also compromise the patient's ability to participate in language tasks, further restricting the utility of awake procedures (Hervey-Jumper et al., 2015; Kram et al., 2025). Awake craniotomy also requires speech pathology or neuropsychology teams in addition to experienced neuro-anesthetists and electrophysiology personnel, which increases the resource burden.

In patients where awake craniotomy is contraindicated, the prospect of AF-CCEP under general anesthesia represents a viable alternative for monitoring a critical language pathway, allowing objective assessment of the AF without requiring patient cooperation. Whilst there appears to be a degree of electrophysiological variability in terms of signal quality and reproducibility associated with general anesthesia in contrast to awake procedures (Giampiccolo et al., 2021; Kim et al., 2022), there are sufficient findings to substantiate continued investigation of the technique as a reliable adjunct to conventional DCS paradigms.

In the present study, we report on our initial experience with AF-CCEPs and AF-ACEPs in a retrospective and heterogeneous cohort of patients undergoing resection of supratentorial tumors involving or adjacent to the AF, and relate quantitative electrophysiological data with World Health Organization (WHO) tumor grade, anatomic tumor location, recording montage configuration, anesthesia paradigms and post-operative outcomes.

## 2 Methods

### 2.1 Patient population

Patients undergoing supratentorial tumor resection at several major tertiary institutions between June 2024 and September 2025 were enrolled in the study. Inclusion criteria were: (1) pathologically suspected glioma, and (2) tumor location adjacent to language-eloquent cortex or subcortical white matter pathways involving the AF. Patients were excluded from AF-CCEP analysis if preoperative neurological assessment revealed severe aphasia or other language deficits that precluded reliable baseline comparison. Patients with intraoperative complications (e.g., seizures, hemodynamic instability, or significant cortical hemorrhage) that could interfere with signal interpretation were also omitted. Finally, patients for whom AF-CCEPs could not be reliably elicited due to anatomical limitations, suboptimal electrode placement, or inadequate stimulation response were excluded from subgroup analyses.

All patients underwent preoperative MRI including T1-weighted, fluid-attenuated inversion recovery (FLAIR), and diffusion tensor imaging (DTI) sequences. Tractography of the arcuate fasciculus and other language-relevant pathways was performed in Brainlab Elements (Brainlab AG, Munich, Germany) using deterministic or probabilistic streamline fiber tracking based on a continuous tracking (FACT) algorithm (Catani et al., 2002; Ille et al., 2018). Streamlines were propagated along the principal diffusion direction until encountering a stopping criterion, defined as fiber angulation >30° or a fractional anisotropy (FA) value below threshold. Bidirectional tracking was performed from each seed point to minimize premature termination. Reconstructed fibers were visualized in a 3D environment with standard direction-encoded color mapping (Catani et al., 2002).

When available, rTMS-positive cortical language sites were used to generate seed regions of interest (ROIs; Ille et al., 2018; Sollmann et al., 2020). These were co-registered to anatomical MRI and expanded with a uniform 5-mm radius to account for spatial variability. In cases without rTMS data, anatomical ROIs were placed based on established perisylvian language regions (Catani et al., 2005; Sarubbo et al., 2016). FA and minimum fiber length constraints were adapted from validated language-tract reconstruction protocols (Ille et al., 2018; Sollmann et al., 2020). Final tractography was reviewed by both the neurosurgical and neurophysiology teams to confirm anatomical plausibility and operative relevance prior to mapping and resection.

### 2.2 Non-invasive pre-surgical mapping

Where possible, patients were referred for nTMS pre-surgical motor and language mapping (Nexstim OYJ, Helsinki, Finland) with structural T1 MRI. If fMRI was available, the study's clinical neuroscientist conducting nTMS (R.P·H) was blinded to the results. The standardized nTMS protocol incorporated bilateral resting motor threshold assessments, upper and lower limb motor cortex mapping, and language mapping via repetitive TMS (rTMS) stimulation for the assessment of lateralization and localization of suspected eloquent cortical regions associated with peritumoral cortex. nTMS-language eloquence was characterized as low, mild, or high levels of evidence supporting lateralization or localization, subjected to language-positive rTMS clustering within cortical parcellations. Positive motor and language nTMS seed points were exported for analysis in Brainlab Elements (Brainlab AG, Munich, Germany) to seed patient-specific DTI tractography of the AF, which informed intraoperative electrode placement and subcortical stimulation targets for AF-CCEP and AF-ACEP acquisition.

### 2.3 Intraoperative electrophysiological mapping

All electrophysiological testing was performed by a single experienced clinical neuroscientist (R.P·H.) across all participating centres, ensuring consistency of stimulation parameters, electrode placement, signal acquisition, and intraoperative interpretation throughout the cohort.

In awake procedures, electrophysiological mapping incorporated bipolar low-frequency (50 Hz) direct cortico-subcortical stimulation (bDCS) for the assessment of language eloquence with continuous electrocorticography (ECoG) and electromyography (EMG) for the mitigation of after-discharges and focal or generalized seizure activity. If motor mapping and monitoring was indicated, monopolar high-frequency (250 Hz) direct cortico-subcortical stimulation (mDCS) was employed along with the same parameters elicited via a subdural strip electrode positioned toward the motor cortex for subdural motor evoked potential (sMEP) monitoring.

**Fig. 1:**
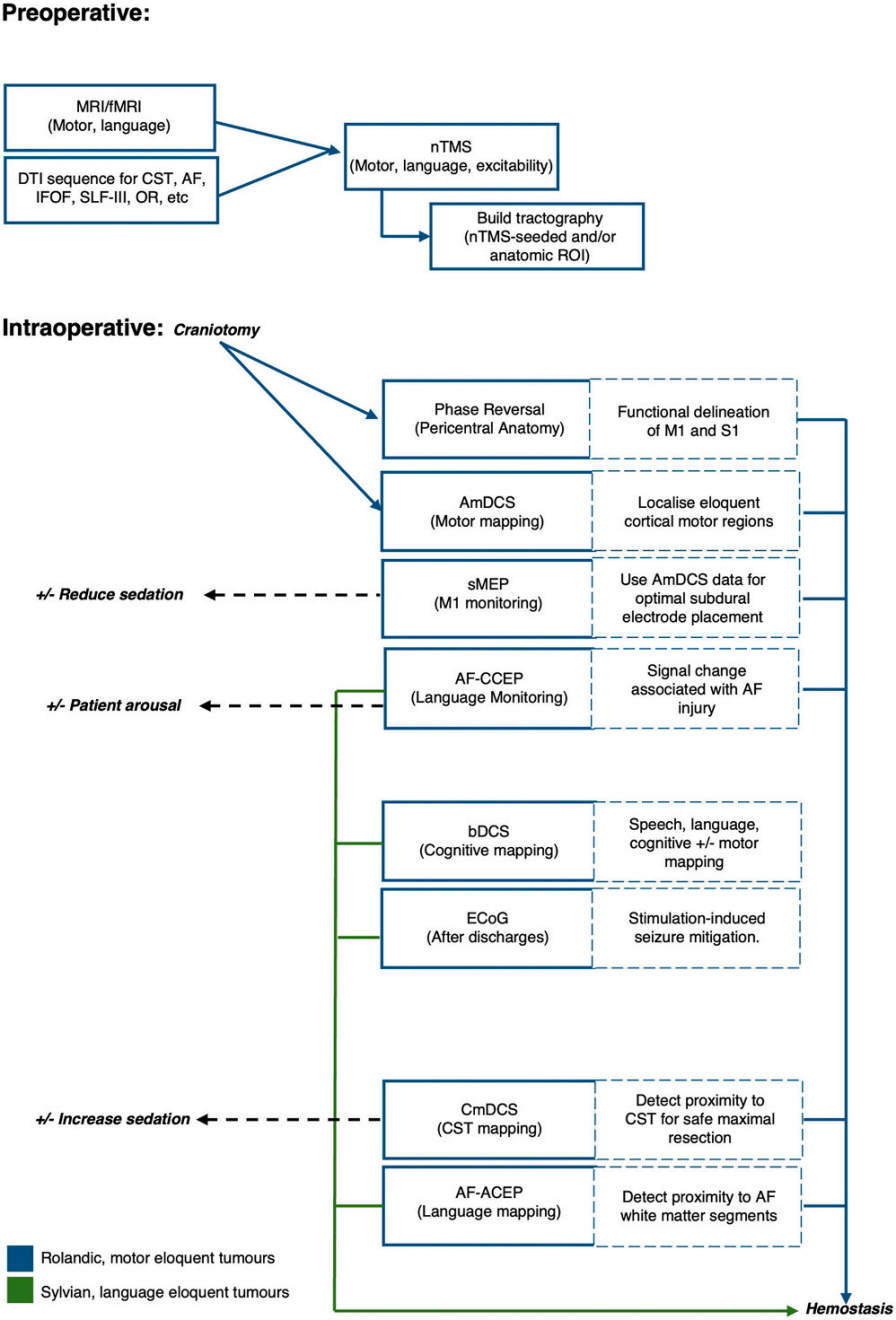
Schematic illustration detailing preoperative and intraoperative mapping workflow to incorporate AF-CCEP and AF-ACEP with contemporary modalities.

AF-CCEPs were elicited via monophasic, constant-current, single-pulse stimulation through a subdural strip electrode with four contacts (inomed Medizintechnik GmbH, Emmendingen, Germany, or WISE Srl, Milan, Italy) positioned toward the inferior frontal gyrus, pars opercularis and/or pars triangularis. Responses were recorded from the middle or posterior superior temporal gyrus using a second subdural strip electrode. Both electrodes were positioned under guidance from stereotactic navigation (Brainlab AG, Munich, Germany). If direct exposure to these regions was not permitted due to a restrictive craniotomy, electrodes were introduced subdurally. Electrode configurations included referential (referenced to the contralateral mastoid) and bipolar (adjacent inter-electrode pairs) montages. When clinically indicated, an additional electrode was positioned over the primary motor cortex for sMEP monitoring (Fig. 1).

Stimulation was delivered via adjacent pairs on the anterior electrode (e.g., CCEP1–2, CCEP3–4) using monophasic single pulses with alternating polarity (0.5 ms pulse width, 10–20 mA intensity, 1.1–1.3 Hz frequency). Valid AF-CCEP and AF-ACEP responses were defined by a clearly identifiable N1 deflection with consistent morphology across trials, occurring within 10–30 ms post-stimulation. The earliest consistent positive deflection (N1) was identified post-stimulation; later components (i.e., P1, N2) were documented where present but not included in primary statistical analysis. If a negative deflection was observed at the suspected N1 onset latency, the signal was inverted. N1 amplitude and latency were measured relative to stimulus onset. Each stimulation site was tested with at least two trials of 30 averages. If no after-discharges or epileptogenic activity were observed at 10–20 mA, the higher stimulation amplitude was accepted as a standardized intensity within our cohort. This stimulation and verification paradigm has been described previously in one of our awake patients (Hamer and Poonnoose, 2025). To confirm physiological rather than passive or volume-conducted responses, low-intensity ‘zero-tests’ were performed at sub-threshold levels to verify loss of reproducibility and absence of N1.

The intraoperative alarm criterion for clinically significant AF-CCEP signal change was defined as a reduction in N1 peak amplitude exceeding 50% from baseline, consistent with previously reported thresholds associated with postoperative language morbidity (Yamao et al., 2017; Kim et al., 2022). Latency prolongation was documented but not used as a primary alarm criterion given its relative stability across our cohort and in prior literature.

Recordings were bandpass filtered at 10–1500 Hz (digital), with hardware filtering between 30 Hz (high-pass) and 5 kHz (low-pass). Data were sampled at 20 kHz. Trials with noise or artifact were excluded. All data were analysed offline in NeuroExplorer (inomed Medizintechnik GmbH, Emmendingen, Germany). A notch filter was not applied during signal acquisition, as this was a deliberate methodological choice to avoid distortion of N1 waveform morphology; trials contaminated by mains noise were excluded during offline artifact rejection.

AF-ACEPs were recorded in a subset of patients (*n* = 10) using the same stimulation parameters but delivered via a bipolar ball-tip probe and recorded at posterior electrode positioned at the middle or posterior temporal gyrus. This subset included two patients where AF-ACEPs were an exclusive adjunct to bDCS language mapping without AF-CCEPs due to limited exposure permitted by the craniotomy for the anterior stimulating electrode. Stimuli were delivered directly to subcortical sites within or around the tumor cavity, informed by nTMS-seeded language fiber bundle or anatomic DTI tractography of the AF. Stimulation was first directed beyond and then within the expected tract trajectory to confirm functional specificity. For AF-ACEPs, >10 μV peak amplitude with biphasic morphology was considered positive, consistent with previous studies where amplitudes in the 10–20 μV range are reliably distinguishable from noise under similar recording and anesthetic conditions (Giampiccolo et al., 2021). Subcortical AF-ACEP findings were registered via stereotactic navigation and assessed in conjunction with nTMS-seeded or anatomic DTI reconstruction of the AF.

### 2.4 Anesthesia

Patients with a preoperative history of seizure activity were maintained on their established anti-epileptic regimen perioperatively. Those without reported seizure activity were commenced on levetiracetam 500 mg three days prior to surgery, with anti-epileptic therapy continued for a minimum of three months postoperatively in all cases.

For patients undergoing asleep craniotomy, anesthesia was maintained using propofol-remifentanil total intravenous anesthesia (TIVA). Halogenated volatile agents were avoided or restricted to a maximum of 0.5 minimum alveolar concentration (MAC), in accordance with their recognized suppressive effects on cortical excitability and potential confounding influence on intraoperative electrophysiological monitoring. Neuromuscular blockade was employed to facilitate endotracheal intubation but was subsequently avoided or pharmacologically reversed prior to the initiation of neurophysiological monitoring.

During awake craniotomies, an asleep-awake-asleep protocol was employed in the majority of cases. Anesthesia was induced and maintained during the craniotomy and closure phases using propofol-remifentanil TIVA, supplemented by dexmedetomidine as an adjunctive sedative agent. Dexmedetomidine, a selective alpha-2 adrenoceptor agonist, was administered as a loading infusion of 0.5 to 1.0 μg/kg over ten minutes prior to incision, followed by a continuous maintenance infusion of 0.2 to 0.7 μg/kg/h for the duration of the procedure. During the awake mapping phase, propofol and remifentanil infusions were titrated down or discontinued to permit full patient cooperation with language, motor, and cognitive tasks, while dexmedetomidine was continued as a background infusion to attenuate procedural anxiety and optimize patient tolerability. Airway management during the sedated phases was achieved via laryngeal mask airway or monitored anesthesia care, determined according to individual patient factors and the judgment of the attending anesthetist.

Intraoperative seizure management was governed by a predefined protocol applied uniformly across the cohort. After-discharges or focal seizure activity identified via ECoG were managed with cortical irrigation using ice-cold Ringer's lactate solution. Generalized seizure events were treated with an intravenous propofol bolus of 0.5 to 1.0 mg/kg, with temporary suspension of the awake phase and reversion to a sedated state undertaken where clinically indicated.

### 2.5 Statistical analyses

Data were analysed in JASP (version 0.19.3, Amsterdam, NL). Distributions were assessed with Shapiro-Wilk. As N1 amplitude data were non-normal, the Wilcoxon signed-rank test was the primary test for paired pre-post comparisons; paired *t*-tests are reported for completeness. For independent groups (e.g., anesthesia, grade), Mann-Whitney *U* tests were used. The Wilcoxon test excludes pairs with zero change; therefore, the effective sample size (n) varies by subgroup and is reported with each result. Two-sided α = 0.05 was used. Subgroup analyses and correlations were prespecified but exploratory, and *p* < 0.001 is reported instead of 0.000.

For each channel, baseline and final N1 peak amplitudes were treated as paired observations. Distributions of amplitude and change scores (final-baseline) were inspected visually and with the Shapiro-Wilk test, demonstrating marked positive skew and heavy tails in most subgroups. Accordingly, Wilcoxon signed-rank tests were used for paired comparisons, and results are summarized primarily using median values, ranges, and median change (Δ). Mean (SD) values are also provided in the descriptive table for completeness, but inference is based on the non-parametric analyses.

Tumors were classified using the 2021 WHO CNS grading system, and subgroup comparisons were performed by tumor grade (lower grade glioma, higher grade glioma), anatomical location (frontal, parietal, temporal), montage configuration (bipolar, referential), and anesthesia paradigm (awake, asleep). Pre- and post-operative language assessments were conducted by speech pathologists, neuropsychologists and/or the consultant neurosurgeon.

This study was approved by the St Vincent's Hospital Melbourne Human Research Ethics Committee (292/24). As a retrospective clinical audit, it was conducted under the National Mutual Acceptance scheme with local governance approvals in place for participating institutions. No data generated within this study informed operative decision making due to the emerging evidence base of the technique.

## 3 Results

### 3.1 Clinical data

Nineteen patients undergoing suspected language-eloquent tumor resection were included. Demographics are presented in Table 1. Seventeen patients contributed AF-CCEP data and ten contributed AF-ACEP data (including two ACEP-only cases). AF-ACEP mapping was not performed in all patients due to one or more of the following: restrictive craniotomy precluding safe probe access to the resection cavity, absence of a clearly defined subcortical resection margin at the time of monitoring, or surgeon preference for awake subcortical DCS as the primary language mapping adjunct. Patients in whom AF-ACEP was performed did not differ systematically from those without AF-ACEP mapping with respect to tumor grade, location, or anesthesia paradigm, though formal comparison was limited by small subgroup sizes.

**Table 1:** Patient cohort demographic statistics.

**N**	**19**
CCEP patients⁎⁎	17
ACEP patients	10
**Gender**	
Male	13 (68.4%)
Female	6 (31.6%)
Median age (range)	34 (4–80)
**Tumor Location**	
Frontal	10 (52.6%)
Parietal	5 (26.3%)
Temporal	4 (21.1%)
**WHO tumor grade**	
LGG	12 (63.2%)
HGG	7 (36.8%)
**Anesthesia**	
Awake	10 (52.6%)
Asleep	9 (47.4%)
**nTMS mapping**	9 (47.4%)

⁎⁎ ACEP-only in 2 patients.

The cohort included 13 males (68%) and 6 females (32%), median age 34 years (range 4–80). Tumor locations were frontal (52.6%), parietal (26.3%), and temporal (21.1%). Twelve patients (63%) underwent resection for lower-grade gliomas (LGG; WHO grade 1–2) and seven (37%) for higher-grade gliomas (HGG; WHO grade 3–4). One patient presented with intraventricular meningioma originally with a differential diagnosis of higher grade glioma and involving perisylvian corticectomy, and was classified as LGG for analysis with nTMS suggesting ipsilesional language lateralisation. Procedures were split between awake (52.6%) and asleep (47.4%) craniotomy. nTMS was performed in nine patients (47.4%) for preoperative assessment of language lateralization and localization. Histopathology and anatomical details are presented in Table 2.

**Table 2:** Patient cohort with tumor histology, tumor location, parcellation and anesthesia type.

Age	Sex	Tumor Histology	Hemisphere	Tumor Lobe	Tumor Parcellation	Anesthesia	IONM	nTMS	Languages Spoken	Signal Reduction	Postoperative Language
41	M	WHO2 oligodendroglioma IDH mutant 1p/19q co-deleted	Left	Parietal	IPL	Asleep	CCEP, mDCS, sMEP, ECoG, EMG	Y; contralesional lateralisation	Monolingual	> 80%	Aphasia
31	M	WHO 2 astrocytoma iDH mutant	Left	Temporal	PrG	Awake	ACEP, bDCS, ECoG, EMG	N	Bilingual	Nil	Minor transient aphasia
57	M	WHO 2 astrocytoma	Left	Frontal	IFG	Awake	ACEP, bDCS, ECoG, EMG	N	Bilingual	Nil	No change
58	M	WHO 4 glioblastoma	Left	Parietal	PoG	Asleep	CCEP, bDCS, ECoG, mDCS, sMEP, EMG	Y; ipsilesional lateralisation	Monolingual	Nil	No change
29	M	WHO 4 high grade glioma	Left	Frontal	MFG	Awake	CCEP, bDCS, ECoG, mDCS, sMEP, EMG	N	Bilingual	Nil	No change
27	F	WHO2 oligodendroglioma	Left	Temporal	MTG	Awake	CCEP, bDCS, ECoG, EMG	N	Monolingual	Nil	No change
36	M	WHO2 oligodendroglioma IDH mutant 1p/19q co-deleted	Right	Frontal	SFG	Awake	CCEP, ACEP, bDCS, ECoG, mDCS, sMEP, EMG	Y; ipsilesional lateralisation	Monolingual	Nil	No change
34	M	WHO2 astrocytoma	Left	Parietal	aSMG	Asleep	CCEP, mDCS, sMEP, ECoG, EMG	Y; ipsilesional lateralisation	Monolingual	Nil	No change
80	M	WHO4 glioblastoma, TERT promotor mutation, IDH wild type	Left	Frontal	IFG	Awake	CCEP, ACEP, bDCS, ECoG, mDCS, sMEP, EMG	Y; ipsilesional lateralisation	Bilingual	Nil	No change
22	M	WHO4 glioblastoma IDH mutant	Left	Temporal	MTG	Asleep	CCEP, ACEP, mDCS, sMEP, ECoG, EMG	N	Monolingual	Nil	No change
34	F	WHO4 glioblastoma	Left	Temporal	STG	Asleep	CCEP, mDCS, sMEP, ECoG, EMG	N	Monolingual	Nil	No change
34	F	WHO4 astrocytoma IDH mutant	Right	Frontal	SFG	Awake	CCEP, ACEP, bDCS, ECoG, EMG	Y; ipsilesional lateralisation	Bilingual	Nil	No change
4	M	Paediatric diffuse lower grade glioma	Left	Frontal	SFG	Asleep	CCEP, mDCS, sMEP, ECoG, EMG	N	Monolingual	Nil	No change
28	F	WHO2 astrocytoma	Left	Parietal	aSMG	Awake	CCEP, ACEP, bDCS, ECoG, EMG	Y; ipsilesional lateralisation	Bilingual	Nil	No change
31	M	WHO2 oligodendroglioma	Left	Frontal	MFG	Awake	CCEP, ACEP, bDCS, ECoG, mDCS, sMEP, EMG	Y; ipsilesional lateralisation	Bilingual	Nil	No change
34	M	WHO2 oligodendroglioma	Left	Frontal	IFG	Asleep	CCEP, ACEP, EMG, ECoG	N	Bilingual	Nil	Minor transient expressive dysphasia
59	F	WHO3 oligodendroglioma	Left	Frontal	IFG	Awake	CCEP, bDCS, ECoG, EMG	N	Bilingual	Nil	No change
46	F	WHO1 intraventricular meningioma	Left	Parietal	–	Asleep	CCEP, mDCS, sMEP, ECoG, EMG	Y; ipsilesional lateralisation	Bilingual	Nil	Minor transient aphasia
62	M	WHO2 astrocytoma	Left	Frontal	MFG	Asleep	CCEP, mDCS, sMEP, ECoG, EMG	N	Bilingual	Nil	No change

### 3.2 AF-CCEP properties

A total of 822 AF-CCEPs and 134 AF-ACEPs were recorded intraoperatively. Of these, 133 CCEPs were matched pre- and post-resection for statistical comparison of peak N1 amplitude and latency (Table 3), in addition to 37 AF-ACEPs. As amplitude distributions were non-normal (Shapiro–Wilk W = 0.871, *p* < 0.001), Wilcoxon signed-rank testing was the primary analysis. Wilcoxon excludes pairs with zero change, as such effective subgroup cohorts vary as reported. Mean and standard deviation data is provided in Table 3, however as this test is based on the median of the paired differences, median values and median change (Δ) are reported as the primary summary measures (Table 4). No after discharges or seizure activity was observed within this cohort.

**Table 3:** AF-CCEP and AF-ACEP peak amplitude and onset latency descriptive statistics.

		Baseline		Final	
N1 Peak Amplitude (uV)	N	*Mean (SD)*	*Median (Range)*	*Mean (SD)*	*Median (Range)*
Total	133	53.21 (±64.35)	29.4 (2.6–411.1)	60.23 (±57.87)	41.8 (0.56–339.7)
Lower-grade tumors	100	54.40 (±72.09)	27.6 (2.6–411.1)	57.92 (±57.99)	36.85 (0.56–339.7)
Higher-grade tumors	33	49.63 (±31.38)	41.9 (13.1–141.9)	66.40 (±57.98)	51.3 (9.6–272.5)
Bipolar Montage	37	41.32 (±59.10)	22.0 (2.6–273.2)	39.28 (±46.95)	26.0 (0.56–241.1)
Referential Montage	96	57.80 (±65.98)	37.6 (5.6–411.1)	68.09 (±59.83)	48.85 (9.5–339.7)
Frontal	72	67.02 (±81.13)	35.15 (6.1–411.1)	75.22 (±62.34)	51.15 (9.6–339.7)
Parietal	41	35.10 (±26.71)	27.1 (2.6–106.7)	34.28 (±25.56)	30.0 (0.56–117.4)
Temporal	20	40.63 (±32.74)	27.15 (6.8–134.9)	68.46 (±73.99)	40.35 (9.6–272.5)
Asleep	82	62.69 (±77.99)	29.95 (2.6–411.11)	71.23 (±70.03)	43.85 (0.56–339.7)
Awake	51	37.97 (±26.36)	28.70 (6.8–141.9)	45.13 (±29.47)	37.30 (9.6–123.5)
**N1 Onset Latency (ms)**					
Total	133	16.02 (±4.49)	15.3 (9.5–32.8)	15.74 (±4.23)	15.2 (8.9–26.7)
Lower-grade tumors	100	15.30 (±4.19)	14.6 (9.5–32.8)	15.66 (±4.06)	15.3 (8.9–26.7)
Higher-grade tumors	33	18.22 (±4.74)	18.5 (9.5–29.8)	15.98 (±4.70)	14.3 (10.3–26.4)
Bipolar Montage	37	16.44 (±5.10)	15.8 (10.2–28.1)	16.47 (±4.07)	15.4 (9.3–26.4)
Referential Montage	96	15.86 (±4.26)	15.2 (9.5–32.8)	15.47 (±4.28)	14.7 (8.9–26.7)
Frontal	72	16.68 (±5.11)	16.4 (9.5–32.8)	15.09 (±4.04)	14.2 (8.9–26.7)
Parietal	41	14.69 (±3.74)	13.4 (10.2–28.0)	15.38 (±4.27)	14.6 (9.3–25.5)
Temporal	20	16.40 (±2.78)	17.3 (9.5–19.3)	18.45 (±3.78)	16.6 (15.2–26.4)
Asleep	82	15.32 (±3.98)	14.7 (10.2–32.8)	15.60 (±4.10)	15.2 (8.9–26.4)
Awake	51	17.15 (±5.06)	16.7 (9.5–29.8)	15.94 (±4.42)	15.3 (10.3–26.7)
**AF-ACEP Peak amplitude (uV)**	37	146.34 (±94.33)	113 (9.5–407.3)		
**AF-ACEP Onset latency (ms)**	37	19.43 (±7.92)	19.8 (9.4–39.0)

**Table 4:** AF-CCEP peak amplitude subgroup statistical analyses.

N1 Peak Amplitude (uV)	n (pairs)	Baseline Median (Range)	Final Median (Range)	Median Δ (μV)	Wilcoxon z	p-value
Total⁎⁎	121	29.4 (2.6–411.1)	41.8 (0.56–339.7)	5.8	−3.30	**0.001**
LGG (WHO1–2)	88	27.6 (2.6–411.1)	36.9 (0.56–339.7)	5.3	−2.48	**0.013**
HGG (WHO3–4)	33	41.9 (13.1–141.9)	51.3 (9.6–272.5)	7.1	−2.30	**0.021**
Bipolar montage	33	22.0 (2.6–273.2)	26.0 (0.56–241.1)	0.2	−0.47	0.646
Referential montage	88	37.6 (5.6–411.1)	48.9 (9.5–339.7)	6.6	−3.49	**<0.001**
Frontal tumors	60	35.2 (6.1–411.1)	51.2 (9.6–339.7)	5.5	−2.37	**0.018**
Parietal tumors	41	27.1 (2.6–106.7)	30.0 (0.56–117.4)	0.8	−0.14	0.905
Temporal tumors	20	27.2 (6.8–134.9)	40.4 (9.6–272.5)	14.7	−3.26	**0.001**
Asleep procedures	70	29.9 (2.6–411.1)	43.9 (0.56–339.7)	5.1	−2.25	**0.025**
Awake procedures	51	28.7 (6.8–141.9)	37.3 (9.6–123.5)	6	−2.33	**0.02**

⁎⁎ Wilcoxon excludes ties/zeros or incomplete pairs.

**Fig. 2:**
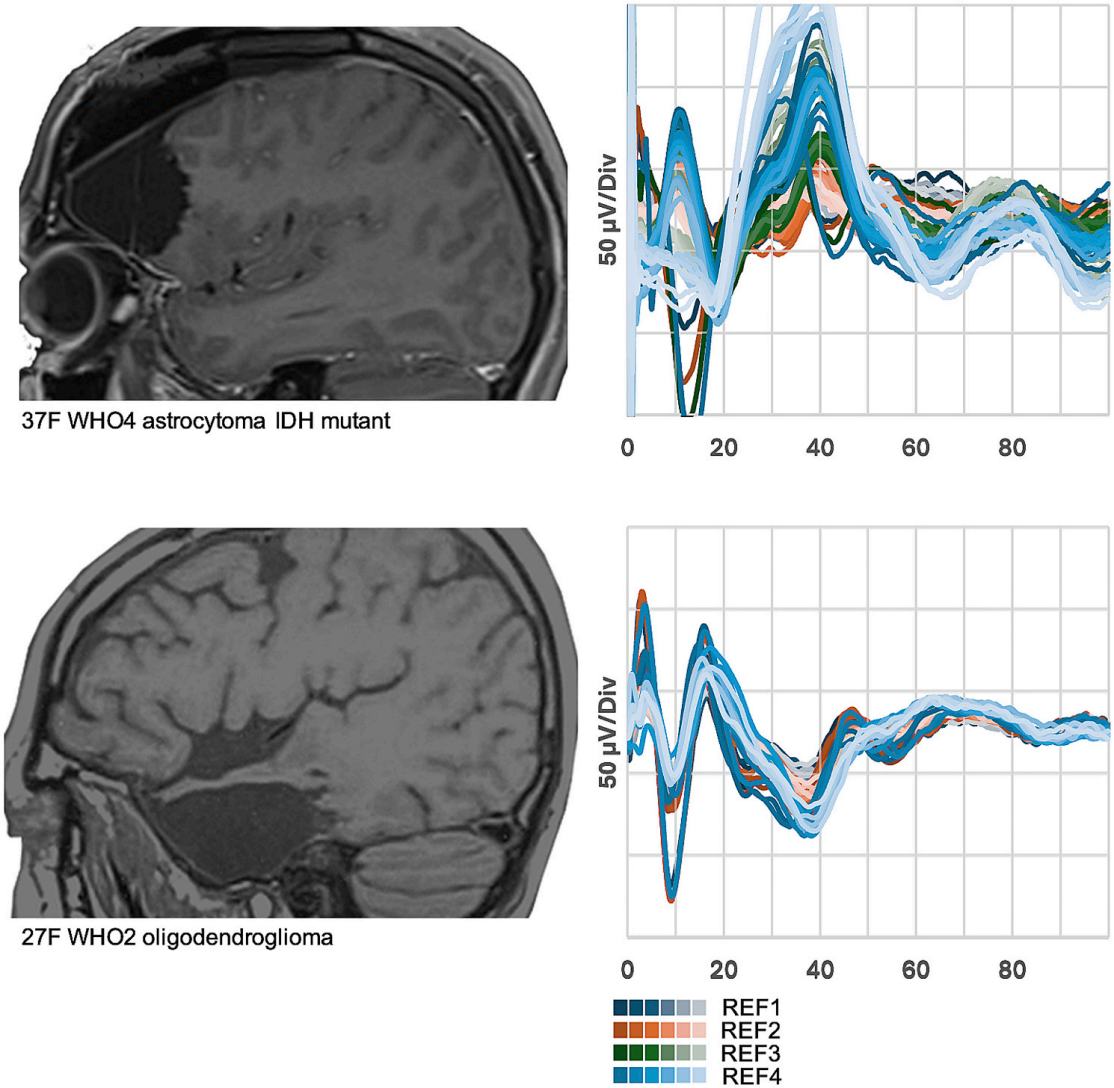
Illustrative comparison of AF-CCEP data indicating one patient with N1 peak amplitude variability throughout resection (top), and another patient with minimal changes throughout resection (bottom).

Across the cohort, AF-CCEP N1 peak amplitude increased significantly following resection (z = −3.30, *p* = 0.001; Table 4,  example in Fig. 2). N1 onset latency was stable overall (baseline 16.02 ± 4.49 ms; final 15.74 ± 4.23 ms) and did not show consistent change across subgroups. One patient exhibited a clinically significant AF-CCEP reduction under general anesthesia and developed postoperative conduction aphasia (Fig. 4).

Briefly, this patient underwent asleep craniotomy for a left parietal WHO2 oligodendroglioma IDH mutant with contralesional language lateralisation on nTMS. During deep posterior resection, near-complete loss of bipolar AF-CCEP signal was observed with only modest reduction in referential montage channels. The patient developed postoperative conduction-type aphasia with approximately 80% recovery at twelve-month follow-up. Postoperative diffusion MRI demonstrated focal fractional anisotropy attenuation and streamline discontinuity along the posterior AF, consistent with iatrogenic dissection.

Subgroup analyses demonstrated post-resection N1 amplitude increases in LGG (*n* = 88, z = 1196, *p* = 0.013) and HGG (*n* = 33, z = 152, *p* = 0.021). By montage, referential recordings increased (z = 974, p < 0.001) whereas bipolar recordings did not (*p* = 0.646). Anatomically, frontal (z = 593.5, *p* = 0.018) and temporal (z = 14, p = 0.001) tumors showed significant increases, whereas parietal tumors did not (*p* = 0.905), supporting AF-adjacent network effects rather than a nonspecific global change. Similar increases were observed under both anesthesia conditions (general: z = 753.5, *p* = 0.025; awake: z = 396.5, *p* = 0.020). Awake patients showed lower baseline amplitudes but experienced robust post-resection increases. Asleep patients exhibited higher amplitudes overall. Amplitude distributions in nearly all subgroups violated normality (Shapiro–Wilk *p* < 0.05), supporting the use of non-parametric tests. As all subgroup analyses were prespecified but exploratory, uncorrected *p*-values across multiple comparisons should be interpreted with appropriate caution and are considered hypothesis-generating rather than confirmatory.

### 3.3 AF-ACEP properties

AF-ACEPs (*n* = 37) demonstrated mean amplitude 146.3 μV (median 113.0; range 9.5–407.3) and mean latency 19.43 ms (median 19.8; range 9.4–39.0). Amplitudes were higher under general anesthesia vs awake (U = 208.0, *p* = 0.035) and in HGG vs LGG (U = 244.0, *p* = 0.010). Temporal tumors trended toward larger and/or faster ACEPs than frontal (amplitude U = 81.0, *p* = 0.094). Montage effects on amplitude were not significant (U = 142.0, *p* = 0.987). Amplitude and latency were inversely related (ρ = −0.324, *p* = 0.050); age correlated with smaller amplitudes (ρ = −0.313, *p* = 0.060) and longer latencies (ρ = 0.503, *p* = 0.002). Positive ACEPs were co-localized with nTMS-seeded or anatomic-DTI regions, confirming proximity, whereas negative or low-amplitude responses were more distant, however this was not quantified in the present study. An illustrative example of the AF-ACEP protocol including negative, positive, and confirmatory subcortical CST findings are presented in Fig. 4.

## 4 Discussion

While awake craniotomy with DCS remains the gold standard for identifying language-critical regions in neuro-oncology surgery, growing interest has emerged in AF-CCEP and AF-ACEP as adjunct modalities to reduce the risk of post-operative conduction aphasia. Recent studies have demonstrated the reproducibility, spatial specificity, and potential prognostic value of these signals in glioma surgery, and our current findings add to this growing body of evidence by demonstrating that AF-CCEP and AF-ACEP are practicable within a range of tumor grades, anatomical locations, and anesthetic conditions (Matsumoto et al., 2004; Yamao et al., 2017; Yamao et al., 2014; Saito et al., 2014; Nakae et al., 2020; Giampiccolo et al., 2021; Saito et al., 2022; Vega-Zelaya et al., 2023; Seidel et al., 2024; Kamada et al., 2025; Kim et al., 2022).

N1 onset latency values across the cohort demonstrated minimal intraoperative variability, and were not significantly influenced by tumor grade, montage type, or anesthetic condition. These findings reinforce prior research demonstrating that N1 latency is a stable electrophysiological correlate of white matter conduction velocity along intact long-range association tracts such as the AF (Matsumoto et al., 2004; Yamao et al., 2014; Giampiccolo et al., 2021; Kim et al., 2022). The observed latencies within our cohort were generally between 15 and 25 ms, which aligns with conduction times based on tract length and axonal properties (Giampiccolo et al., 2021; Kim et al., 2022; Silverstein et al., 2020), further supported by positive AF-ACEP findings within the resection cavity that fell within these ranges, implying anato-functional concordance.

N1 peak amplitude demonstrated significant amplitude increases following resection, in particular for tumors infiltrating frontal and temporal lobes. Mechanistically, this may be representative of peri-resection hyperexcitability and network-level re-engagement, such as reduced peritumoral GABAergic inhibition, glutamatergic effects, synaptogenesis, edema or impedance changes, and unmasking of polysynaptic pathways (Venkatesh et al., 2019; Aerts et al., 2020; Krishna et al., 2021; Duffau, 2021). Given the curvature and segmentation of the AF, part of the increase may index divergent or polysynaptic activity rather than purely monosynaptic transmission (Matsumoto et al., 2004; Jehna et al., 2017). Variability of AF-CCEP morphology according to tumor histology have also recently been reported (Seidel et al., 2024). Additionally, the recruitment of compensatory or parallel networks in patients with chronic glioma-related plasticity may enhance effective connectivity, particularly in language systems (Ng et al., 2023; Krishna et al., 2021).

**Fig. 3:**
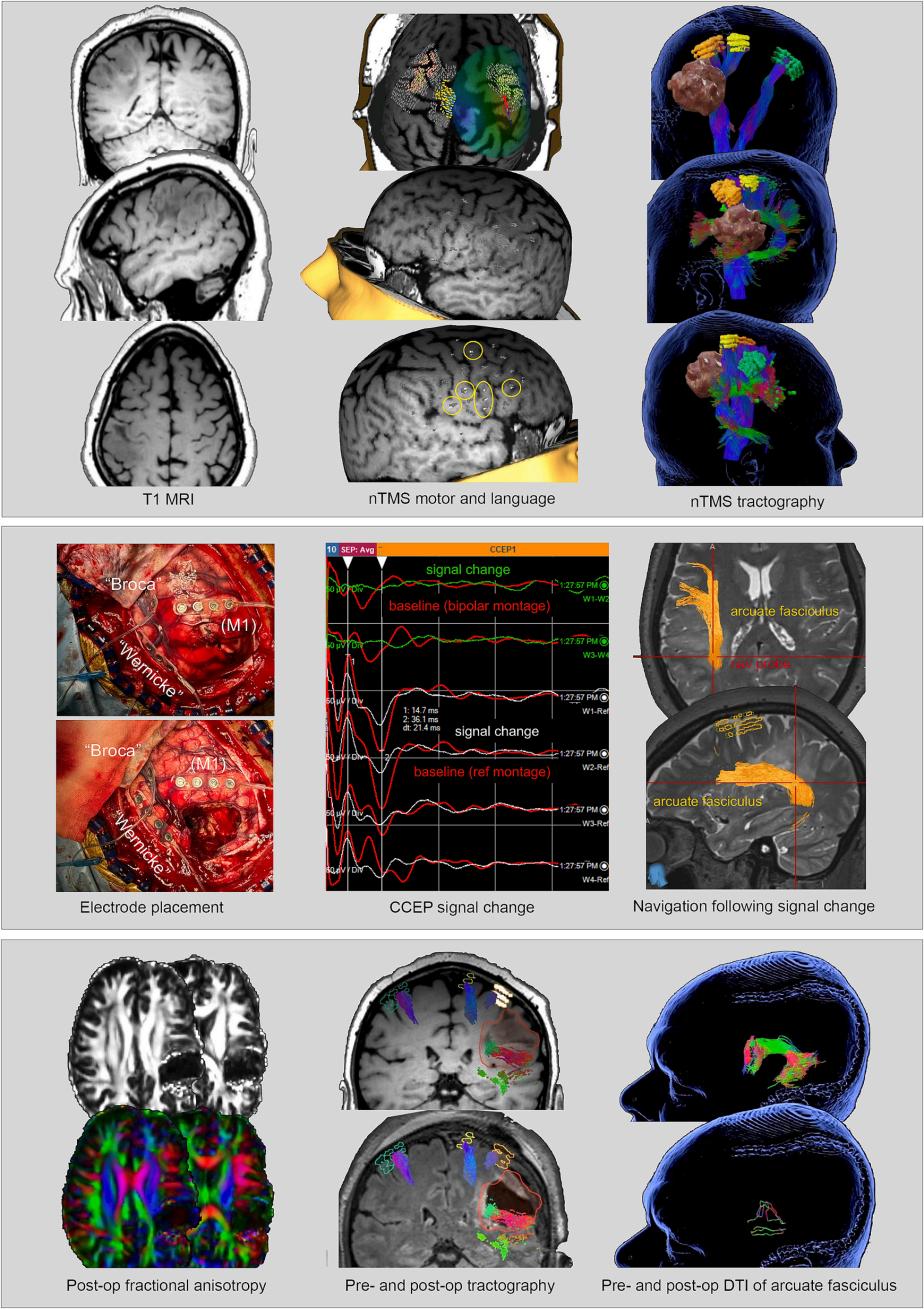
Illustrative example highlighting intraoperative AF-CCEP signal change. (A) Preoperative structural T1 MRI (left) overlaid with nTMS delineating motor and language-positive sites (middle), with corresponding diffusion tractography illustrating the AF and related nTMS language fiber-bundle (right). (B) Intraoperative image of subdural strip electrode placement targeting Broca's and Wernicke's homologues with the primary motor cortex (left), with AF-CCEP traces demonstrating latency and amplitude signal changes across bipolar and referential montages (center), with navigated image guidance confirming proximity of stimulation site to the arcuate fasciculus (right). (C) Postoperative assessments including fractional anisotropy maps (left), co-registered pre- and post-operative tractography showing subtle changes in white matter organization (middle), and targeted DTI of the arcuate fasciculus revealing preserved structural integrity (right).

N1 peak-amplitude reductions of greater than 50% are associated with persistent or permanent impairment (Yamao et al., 2017; Yamao et al., 2021), whereas reductions less than 50% have been associated with transient postoperative language morbidity reported to recover within approximately three months (Kim et al., 2022). We experienced one instance of near-complete bipolar AF-CCEP signal loss associated with significant post-operative conduction aphasia, with referential channels reduced only by approximately 40% (Fig. 3). No other signal change or injury was observed in our cohort.

Preoperatively, this patient's seizure semiology was characterized by preserved speech during generalized events, consistent with atypical or bilateral language representation. This is further supported by reports that speech-related phenomena during seizures have limited but sometimes lateralizing value toward non-dominant onset, and with documented language network reorganization in tumor-related pathology (Ng et al., 2023; Duffau, 2005; Krishna et al., 2021; Rosenow and Lüders, 2001). This was reinforced by comprehensive and un-ambiguous nTMS findings supporting contralesional language dominance, indicating potential lateralization variability rather than excluding ipsilesional dorsal-stream involvement.

Awake craniotomy was not feasible for this patient due to resource limitations, so surgery proceeded under general anesthesia with mapping incorporating AF-CCEPs. During deep resection, dissection near the posterior AF was followed by a near-complete loss of bipolar AF-CCEPs and the patient developed postoperative conduction-type aphasia. Post-operative diffusion MRI with fractional anisotropy maps and co-registered DTI tractography demonstrated focal attenuation and streamline discontinuity along the posterior AF, consistent with mild dissection. In retrospect, adjunctive AF-ACEP mapping might have provided additional tract-level resolution to help avoid AF disruption in this context of atypical lateralization. At twelve month follow-up, this patient experienced approximately 80% recovery of aphasia symptoms in comparison to their respective baseline.

A montage-dependent pattern was observed within our cohort. Referential recordings yielded higher mean amplitudes than bipolar configurations, consistent with previous studies highlighting greater signal capture from spatially broader sources (Matsumoto et al., 2004; Yamao et al., 2014). However, bipolar recordings offered greater spatial resolution and were more resistant to volume conduction artefacts, with characteristics that enhance the precision of intraoperative functional localization important for operative decision-making (Yamao et al., 2014; Nakae et al., 2020). Our single case of postoperative conduction aphasia with near-complete bipolar AF-CCEP loss with only modest referential montage signal change illustrates why recording both montages is prudent to reduce the likelihood of false negative interpretation. It is also worth acknowledging that the AF encompasses a broader network of cortical terminations beyond the classical Broca-Wernicke axis, including middle frontal, premotor, and inferior parietal regions (Giampiccolo et al., 2021). This heterogeneity likely contributes to variability in AF-CCEP morphology observed across our cohort, particularly in patients with frontal and parietal tumors, and should be considered in the design of future prospective studies with standardized electrode placement protocols.

Evidently, AF-CCEP does not prevent iatrogenic dissection. AF-ACEPs within the resection cavity provided complementary insights regarding proximity to the AF that may promote opportunities of safe maximal resection otherwise encumbered by DTI tractography. In the present study, AF-ACEPs were included as an exploratory investigation to consider the feasibility of a relationship between positive electrophysiological findings (i.e., peak-amplitude) and proximity to AF as indicated via stereotactic navigation systems in-situ. Indeed, all positive AF-ACEP data in our limited cohort were associated with proximity to tractography data though a distance-to-tract relationship was not defined and will be investigated in subsequent studies. Interestingly, one positive AF-ACEP signal was generated following stimulation involving the superior frontal gyrus in a large diffuse LGG comprising the frontal lobe. Whether this involved stimulation of FAT fibers and transmitted via the AF is speculative but not implausible (Ookawa et al., 2017). These early observations support the feasibility of AF-ACEPs as a tract-proximal adjunct to awake subcortical DCS mapping, rather than a calibrated distance-to-tract tool, and highlight the emerging potential to refine resection margins in language-eloquent glioma surgery under general anesthesia.

**Fig. 4:**
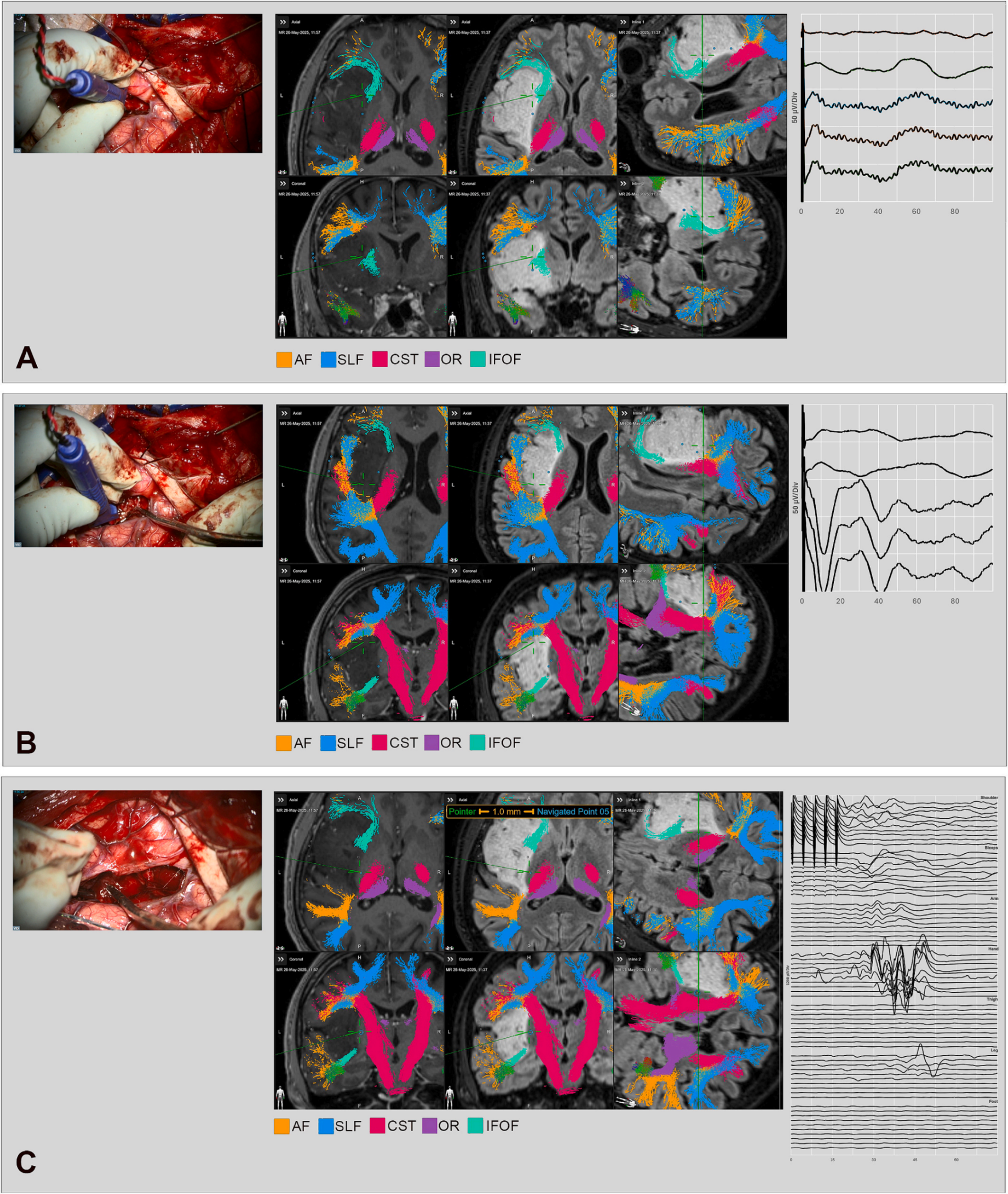
Illustrative examples of intraoperative AF-ACEP protocol. First patient; A: surgeon instructed to apply stimulation away from suspected AF, with accompanying negative AF-ACEP data. B: Surgeon instructed to apply stimulation toward direction of suspected AF at 20 mA, with accompanying polyphasic positive AF-ACEP data. C: Corresponding high frequency monopolar DCS confirming corticospinal tract (pink) activation relative to the arcuate fasciculus (orange). (For interpretation of the references to color in this figure legend, the reader is referred to the web version of this article.)

Variability of AF-ACEP latency data supports the notion that the AF comprises functionally distinct anterior, posterior, and longitudinal subsegments (Catani et al., 2005; Sarubbo et al., 2016), with electrophysiological characteristics (i.e., latency) modulated by the relative positioning of recording electrodes along its trajectory (Rossel et al., 2023). However it is also possible that a high-intensity subcortical stimulation employed during AF-ACEP mapping elicited both orthodromic and antidromic activation, resulting in a robust, polymorphic evoked response (as shown in Fig. 4). This likely reflects the close proximity of the stimulation probe to the divergent cortical terminations of the posterior aspect of the AF, combined with the capacity of subcortical axons to support bidirectional propagation.

A degree of AF-CCEP biosignal variability is expected after iatrogenic manipulation involving the central nervous system. For example, routine modalities such as motor and sensory evoked potentials are typically the most standardized and reproducible, with defined acquisition parameters and widely adopted alarm criteria. In contrast, AF-CCEPs remain an emerging technique whose morphology is more sensitive to electrode placement, montage, and network state, and can also be modulated by anesthetic regimen. Within this context our series showed relative stability of N1 latency consistent with a tract-conduction correlate, whereas N1 amplitude varied post-resection in ways that likely reflect network excitability rather than a consequence of global physiologic or systemic changes (Yamao et al., 2014; Giampiccolo et al., 2021; Kim et al., 2022).

Accordingly we interpret amplitude changes cautiously, and not as artefactual, however we cannot exclude contributions from biophysical factors. Tumor removal alters cavity geometry and tissue conductivity, so we cannot fully dissociate physiological changes from volume-conduction and ‘shunting’ effects. However, several observations argue against a purely physical explanation. For example, N1 latencies remained remarkably stable, amplitude increases were not restricted to channels closest to the cavity, and similar patterns were seen across anesthetic paradigms and montages. We therefore interpret the amplitude changes as reflecting a combination of network-level and biophysical effects and have tempered our mechanistic postulations.

As per recent studies, we agree that more comprehensive data is required in order to generate compelling clinical evidence for routine inclusion of this technique in neuro-oncology surgery, as there is significant complexity associated with the interpretation of such investigational electrophysiological phenomena. Factors such as anesthesia, tissue conductivity, oedema, and electrode placement may introduce variability that complicates threshold-based decision-making, in addition to the electrophysiological thresholds themselves, which are not formally elucidated.

More broadly, combining cortico-cortical and axono-cortical electrophysiological investigations contributes to the possibility of network-level mapping in neuro-oncology surgery. Dynamic tractography that fuses AF-CCEP timing with diffusion modeling further supports this direction and may ultimately permit tract-specific thresholds for resection safety (Yamao et al., 2014; Seidel et al., 2024; Silverstein et al., 2020). Preliminary extensions of these paradigms to non-AF white matter bundles such as the optic radiations (Boëx et al., 2021) and frontal aslant tract (Ookawa et al., 2017) have begun to appear and warrant systematic, prospective evaluation.

Several limitations must also be acknowledged. The sample size, though comparable to similar studies, limits subgroup statistical power and generalizability. The observed distribution of bilingual patients in the awake group reflects individualized clinical decision-making rather than a predetermined criterion; however, this represents a potential confounder between language profile and anesthesia paradigm that prospective studies with standardized allocation criteria should formally address. Nine patients underwent preoperative nTMS mapping, however formal comparison of signal characteristics or outcomes between nTMS and non-nTMS patients was not performed given the small subgroup sizes, though this represents a worthwhile focus for future prospective investigation.

Tumor-AF relationships in this cohort included both infiltration and displacement, however formal quantification of this distinction for each patient was beyond the scope of this retrospective analysis but represents an important variable for future prospective work. While postoperative imaging and language assessments were performed, longer-term follow-up and formalized, standardized language testing would enhance interpretation of functional outcomes. Further, the precise relationship between AF-CCEP signal change and positive AF-ACEP responses with regard to subcortical tract injury remains inferential in most cases, and warrants correlation with various structural and functional neuro-imaging modalities, including a distance-to-tract relationship between positive axono-cortical data and the AF. Future studies should prioritize acquisition parameters and normative AF-CCEP and AF-ACEP values in larger cohorts.

Finally, this was a retrospective, heterogeneous cohort with a modest sample size; subgroup counts were unbalanced, and non-normal amplitude distributions required non-parametric testing, which excludes zero-change pairs and thus reduces the number of paired observations contributing to some comparisons. Multiple subgroup comparisons were conducted without multiplicity adjustment and are therefore exploratory. AF-ACEP analyses were limited to a small subset, without quantitative co-registration of stimulation points to tract distance. Intraoperative factors (i.e., electrode placement, impedance, anesthesia depth) and platform differences may introduce measurement variability, and postoperative language outcomes were not assessed with a uniform, blinded battery at fixed intervals. DTI tractography also has well-described constraints for resolving crossing fibers and small lesions, which may affect anatomical inferences.

## 5 Conclusions

AF-CCEPs and AF-ACEPs constitute robust, reproducible electrophysiological biomarkers of frontotemporal connectivity associated with post-operative AF integrity. N1 onset latency appears to be a stable index of the AF, whereas N1 peak amplitude demonstrated significant post-resection amplitude increases, particularly in referential montages and tumors involving the temporal lobe, suggestive of sensitivity to excitability changes associated with tumor biology, network plasticity or neuroplastic potential, and perioperative factors. While increases in N1 amplitude may reflect peri-resection hyperexcitability, signal loss remains the primary intraoperative indicator of AF injury with postoperative morbidity. Collectively, our findings reinforce the evolving role of AF-CCEP and AF-ACEP as a useful functional adjunct alongside contemporary DCS techniques in neuro-oncological surgery, particularly when awake mapping may not be feasible.

## Author contributions

Conceptualisation, R.P.H.; methodology, R.P.H.; formal analysis, R.P.H.; investigation, R.P.H and A.P.; resources, A.M., C.D., K.B., S.P., A.B. and A.P.; data curation, R.P.H.; original draft preparation, R.P.H.; review and editing, all authors; supervision, A.P. All authors have read and agreed to the published version of the manuscript.

## Declaration of competing interest

RPH is a clinical consultant and distribution partner of Nexstim OYJ (Finland) and inomed medizintechnik gmbh (Germany), whom were not involved in the design or execution of this paper, nor has he received honoraria or incentive of any description for the writing of this manuscript, which was written as part of his academic appointments at the University of Sydney and University of Melbourne. All other authors declare no conflicts of interest.
